# Adiabatic Quantum Computation Applied to Deep Learning Networks

**DOI:** 10.3390/e20050380

**Published:** 2018-05-18

**Authors:** Jeremy Liu, Federico M. Spedalieri, Ke-Thia Yao, Thomas E. Potok, Catherine Schuman, Steven Young, Robert Patton, Garrett S. Rose, Gangotree Chamka

**Affiliations:** 1 Department of Computer Science, University of Southern California, Los Angeles, CA 90089, USA; 2 Information Sciences Institute, University of Southern California, Marina del Rey, CA 90292, USA; 3 Department of Electrical Engineering, University of Southern California, Los Angeles, CA 90089, USA; 4 Computational Data Analytics Group, Oak Ridge National Laboratory, Oak Ridge, TN 37830, USA; 5 Department of Electrical Engineering & Computer Science, University of Tennessee, Knoxville, TN 37996, USA

**Keywords:** deep learning, quantum computing, neuromorphic computing, high performance computing

## Abstract

Training deep learning networks is a difficult task due to computational complexity, and this is traditionally handled by simplifying network topology to enable parallel computation on graphical processing units (GPUs). However, the emergence of quantum devices allows reconsideration of complex topologies. We illustrate a particular network topology that can be trained to classify MNIST data (an image dataset of handwritten digits) and neutrino detection data using a restricted form of adiabatic quantum computation known as quantum annealing performed by a D-Wave processor. We provide a brief description of the hardware and how it solves Ising models, how we translate our data into the corresponding Ising models, and how we use available expanded topology options to explore potential performance improvements. Although we focus on the application of quantum annealing in this article, the work discussed here is just one of three approaches we explored as part of a larger project that considers alternative means for training deep learning networks. The other approaches involve using a high performance computing (HPC) environment to automatically find network topologies with good performance and using neuromorphic computing to find a low-power solution for training deep learning networks. Our results show that our quantum approach can find good network parameters in a reasonable time despite increased network topology complexity; that HPC can find good parameters for traditional, simplified network topologies; and that neuromorphic computers can use low power memristive hardware to represent complex topologies and parameters derived from other architecture choices.

## 1. Introduction

A neural network is a machine learning concept originally inspired by studies of the visual cortex of the brain. In biology, neural networks are the neurons of the brain connected to each other via synapses; accordingly, in machine learning, they are graphical models where variables are connected to each other with certain weights. Both are highly useful in analyzing image data, but practical considerations regarding network topology limit the potential of simulating neural networks on computers. Simulated networks tend to divide neurons into different layers and prohibit intralayer connections. Many-layered networks are called deep learning networks, and the restriction of intralayer connections allows rapid training on graphical processing units (GPUs).

We explain some current limitations of deep learning networks and offer approaches to help mitigate them. For this article we focus on a quantum adiabatic computing approach, which is one of a trio in a larger project to survey machine learning in non-traditional computing environments, though we also describe the other approaches at a high level to offer comparison and context for experiment designs. The second approach uses a high performance computing environment to automatically discover good network topologies, albeit they remain restricted from using intralayer connections. The third approach uses neuromorphic computing as a low-power alternative for representing neural networks. Rather than explicitly choosing one solution or another, these approaches are meant to augment each other. Describing these different approaches necessitates a brief description of various machine learning models and networks including Boltzmann machines (BMs), convolutional neural networks (CNNs), and spiking neural networks (SNNs). Results obtained from CNNs and SNNs, while important to our project, are not the focus of this article and are presented in the appendix.

### 1.1. Boltzmann Machines

A Boltzmann machine is an energy-based generative model of data. BMs contain binary units, and each possible configuration of units is assigned a certain energy based on edge weights. The goal of training is to find edge weights that result in low energy configurations for patterns more likely to occur in data. Since BMs can be represented as Ising models, and because the D-Wave processor is designed to natively solve Ising models, BMs are particularly attractive for our purposes. We tend to view BMs as probabilistic neural networks with symmetrically connected units [1]. BMs are well suited to solving constraint satisfaction tasks with many weak constraints, including digit and object recognition, compression/coding, and natural language processing.

A common algorithm for training BMs exposes a BM to input data and updates the weights in order to maximize the likelihood that the underlying model of the BM reproduces the data set. This method requires computing certain quantities which, due to the specific form of the BM, turn out to be the values of certain correlation functions in thermal equilibrium. However, training is a slow and arduous task if we allow models with unrestricted topology. Connectivity loops slow down the convergence of many algorithms used to estimate thermal equilibrium properties. Simulated annealing is a generic and widely used algorithm to reach this thermal equilibrium, but this remains a slow and expensive process for large networks. This forces us to either use tiny networks or to give up complex topologies, with the latter option leading to the popular choice of using restricted Boltzmann machines (RBMs) [2].

**Figure 1:**
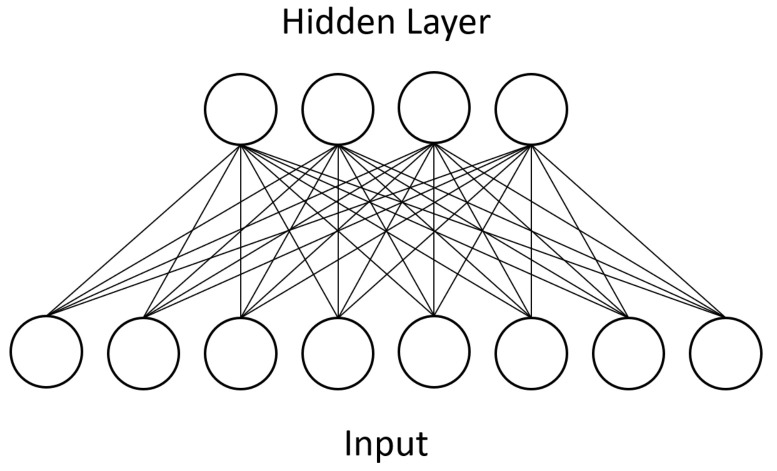
A Boltzmann machine is divided into a visible layer, representing the data input, and a hidden layer, which represents latent factors controlling the data distribution. This diagram shows the restricted Boltzmann machine, or RBM, in which intralayer connections are prohibited. Each connection between units is a separate weight parameter which is discovered through training.

Units in RBMs are categorized as “visible” or “hidden.” During training, the visible units of a RBM represent the input dataset whereas the hidden units represent latent factors that control the data distribution. After undergoing the above training process, an RBM will produce a distribution of visible unit states that should closely match the input dataset. Additionally, only bipartite connectivity between the two types is allowed, which makes parallel computation feasible. Figure 1 shows an example of this bipartite connectivity. Approximation algorithms make training tractable in practice, and RBMs can be stacked together to form deep belief networks (DBNs) [3].

### 1.2. Convolutional Neural Networks

**Figure 2:**
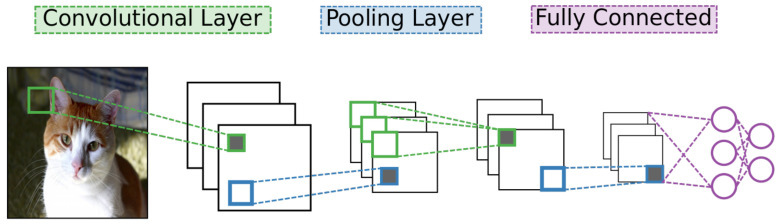
A convolutional neural network is composed of a series of alternating convolutional and pooling layers. Each convolutional layer extracts features from its preceding layer to form feature maps. These feature maps are then down-sampled by a pooling layer to exploit data locality. A perceptron, a simple type of classification network, is placed as the last layer of the CNN.

Of the many designs for deep learning networks, CNNs have become the most widely used for analyzing image data [4]. As with other deep learning networks, CNNs contain many layers of neural units with many connections between different layers but no connections between units of a particular layer. They also use standard stochastic gradient descent and back-propagation combined with labeled data to train. What separates a CNN from other networks are its unique connectivity arrangement and different types of layers. See Figure 2 for a high-level diagram of the CNN architecture.

**Figure 3:**
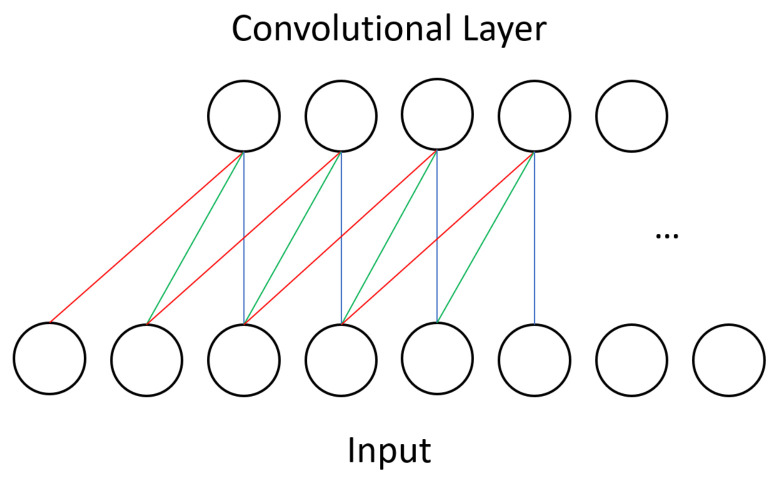
The connectivity in a CNN is sparse relative to the previously shown BM model. Additionally, the set of weights is shared between units, unlike in BMs. In this illustration we symbolize this with the red, green, and blue connections to show that each unit in the convolutional layer applies the same operation to different segments of the input.

One type of layer in CNNs is the convolutional layer. Unlike in other neural networks, a convolutional layer uses a kernel, or small set of shared weights, to produce a feature map of the input to the layer, and many convolutional layers operate in succession. Other networks would typically have every input unit connected to every processing unit in a layer whereas a CNN is satisfied with using convolution to produce sparse connections between layers—see Figure 1 for the dense connectivity of a BM and compare it against the sparse CNN connectivity shown in Figure 3. A kernel captures a certain feature from the input, and convolving a kernel with the data finds this feature across the whole input. For example, a kernel that detects diagonal lines can be convolved with an image to produce a feature map that can be interpreted as identifying all areas of an image that contain diagonal lines.

The second type of layer is the pooling layer. Pooling layers use the many feature maps produced by convolutional layers as input and subsample them to produce smaller feature maps to help take advantage of data locality within images. CNNs use alternating layers of convolutional and pooling layers to extract and abstract image features. Pooling operations makes feature detection in CNNs resilient to position shifts in images [5].

### 1.3. Spiking Neural Networks

SNNs differ from both BMs and CNNs by incorporating the extra dimension of time into how information is processed. BMs and CNNs do not have a sense of time built into their architectures—neural unit activity is iteratively calculated on a layer-by-layer basis. SNNs instead use integrate-and-fire neurons, units that collect activation potential over time and fire or “spike” upon reaching a threshold, after which they cannot fire during what is known as a refractory period. Additionally, synapses in a SNN can include programmable delay components, where larger delay values on the synapse correspond to longer propagation time of signals along that synapse. Additionally, there is not necessarily a division of units into well-organized layers in a SNN, and input is fed to the network over time.

SNNs have great potential in moving away from the traditional implementation of machine learning algorithms on the CPU/memory von Neumann architecture. For example, the CPU/memory model, while useful on many diverse applications, has the drawback of high power requirements. Nature’s biological neural networks have extremely low power requirements by comparison. There are many different ways to implement neuromorphic systems, but one of the more promising device types to include in neuromorphic systems is memristors. Development of memristive technology opens the potential of running spiking neural networks using low power consumption on neuromorphic architectures.

A key challenge associated with SNNs in general and SNNs for neuromorphic systems in particular is determining the correct training or learning algorithm with which to build the SNN. Though there have been efforts to map existing architectures like CNNs to equivalent spiking neuromorphic systems [6,7], there is also potential to develop independent deep learning architectures that exploit the temporal processing power of SNNs.

### 1.4. Challenges

Complex networks pose enormous problems for deep learning, three of which we identify. How we tackle each of these challenges is the basis of our project, where we seek relief from these issues through quantum adiabatic computing, high performance computing, and neuromorphic computing.

The first of these challenges comes from complex network topology in neural networks. By complex network topology we mean bidirectional connections and looping connectivity between neural units, which slow training to a crawl. The training algorithms we know for such complex networks have greater than polynomial runtime, making them effectively intractable and untenable for practical purposes. Deep networks deployed on real-world problems, like the previously discussed CNN architecture, instead impose limitations on network topology. Removing intralayer connections or enforcing strict rules for network topology allows faster and tractable training algorithms to run. However, doing so takes away some of the representational power of the network [8], and these restricted or limited networks do not reflect models found in nature. While tractable models perform remarkably well on specialized classification tasks, we speculate that other more complex and generalized tasks may benefit from the additional representational power offered by complex networks. We believe quantum adiabatic computing offers part of a potential solution through its ability to sample from complex probability distributions such as those generated by neural networks containing intralayer connections.

The second challenge is automatically discovering optimal or near-optimal network hyperparameters and topologies. Hyperparameters in deep learning refer to the model parameters, i.e., the activation function used, the number of hidden units in a layer, the kernel size of a convolutional layer, and the learning rate of the solver. Currently the best deep learning models are discovered by creating, training, testing, and tuning many models on some well-known reference dataset and reporting the best model in the literature. However, if the dataset has not been examined before, it is difficult to know how to tune networks for optimal performance. GPU-based high performance computing provides an opportunity to automate much of this process—to train, test, and evolve thousands of deep learning networks to find optimally-performing network hyperparameters and network topologies.

The last challenge is power consumption, which we can help address through neuromorphic computing. Machine learning’s computational needs have so far been met with power-hungry CPUs and more recently GPUs. The switch from CPUs to GPUs has significantly sped up computation and lowered computation costs, but GPU efficiency in training networks still pales in comparison to the efficiency of biological brains. For an image recognition task, it might take many server farms and a hydroelectric dam to compete with a mundane human brain running on a bit of glucose. Neuromorphic computing offers a potential solution by developing specialized low-power hardware that can implement SNNs approximating trained networks derived from more orthodox architectures.

This article focuses on deep learning’s challenges related to quantum adiabatic computing. Though high performance and neuromorphic computing are an integral part of our project, we move discussions of these topics to the appendix to better fit our focus for this journal, though mentions of both appear as necessary through the rest of the article. Our experiments use the MNIST dataset [9], an image dataset of handwritten digits, and a neutrino particle detection dataset produced by Fermi National Accelerator Laboratory. Next, we will review works related to quantum computing; then we provide our experimental approach, results, and future research.

## 2. Related Work

We look at the current state-of-art quantum computing as it relates to the previously discussed challenges in deep learning. Work related to high performance computing and neuromorphic computing are presented in Appendix A. Though the papers and articles referenced in the appendix are not strictly related to quantum adiabatic computing, they provide context for the larger ORNL project and present existing or proposed systems that can be compared against our own quantum computing efforts.

Feynman first discussed quantum computing within the context of simulation, noting that simulating a quantum system using a classical computer seems to be intractable [10]. Interest in quantum computing surged with the introduction by Shor of a polynomial-time algorithm for factoring integers [11], giving an exponential speedup over the best known classical algorithm and threatening to break most modern encryption systems. As with Turing’s work, these theories for quantum computing were developed before quantum hardware was available. Different models of quantum computing have since been developed in order to explore the power of quantum information processing. In the quantum circuit model (on which Shor’s original algorithm relies), a sequence of unitary transformations are applied to a set of quantum bits (qubits), in a way analogous to the logical gates that are applied to classical bits in classical computing. In the measurement-based quantum computing model [12], a special quantum state is prepared beforehand, and a computation is performed by adaptively applying quantum gates to each qubit and measuring them. In the adiabatic quantum computing (AQC) model [13], a quantum state encoding the solution of a problem is prepared using the adiabatic theorem of quantum mechanics. All three models have the same computational power but also offer different trade-offs. Quantum information is extremely fragile, and any source of noise (like thermal fluctuations, unwanted interactions with an uncontrolled environment, etc.) can destroy the quantum features that are expected to provide a computational speedup. The AQC model has been considered as the most robust implementation, and hence the development of actual devices based on AQC has led that of the other two approaches, both of which are more susceptible to noise and require very large overhead to overcome the effects of that noise. However, currently available devices such as the D-Wave processor are still limited in many aspects, the most important being the fact that they operate at a finite temperature and that the effects of this noise in the performance of the device is still an active area of research. We typically refer to these devices operating at a finite temperature as quantum annealers.

Quantum annealers are in principle designed to solve a particular optimization problem, typically finding the ground state of an Ising Hamiltonian. Unfortunately, thermal fluctuations due to interactions with a finite temperature reservoir, in addition to unwanted quantum interactions with other systems in the environment, tend to kick the system out of its ground state and into an excited state. These unavoidable features make the quantum annealer behave more like a sampler than an exact optimizer in practice. However, this seemingly counterproductive property may be turned into an advantage since the ability to draw samples from complicated probability distributions is essential to probabilistic deep learning approaches such as the Boltzmann machine, which relies heavily upon sampling complex distributions in both training and output. Quantum annealers could then help us overcome the problem of complex topologies mentioned before. BMs in their unrestricted form are impractical to train on classical machines, a fact that led to the development of RBMs that eliminate intralayer edges and introduce bipartite connectivity [2]. Bipartite graphs allow the use of an algorithm known as contrastive divergence that approximates samples from a RBM in linear time, which is a critical tool for the practical usage of BMs because sampling is the core engine for training BMs. Quantum annealing hardware allows us to partially pull back from this bipartite limitation. Quantum annealers provide a novel way to sample richer topologies, and several approaches exploit this feature for different choices of graphs and topologies on D-Wave hardware [14,15,16].

## 3. Approach and Data

Quantum adiabatic computation, high performance computing, and neuromorphic computing differ significantly from each other in how they process data. As such, the amount of data each can support dictated our choice of deep learning problem that could be adapted to each of these three heterogeneous paradigms. At the time that the results were collected, D-Wave supported 1000 qubits (now 2000 qubits), which limited the size of problems we could solve. With this in mind we chose to examine two datasets we refer to as MNIST and neutrino data.

The Modified National Institute of Standards and Technology (MNIST) data set is a well-known collection of hand-written digits extensively studied in the deep learning community. The dataset is composed of images, each of which contains a handwritten digit and an associated label identifying the digit. The digit images are only 28×28=784 pixels, which fits within the 1000 qubit D-Wave hardware and onto HPC and neuromorphic architectures. Our later experiments used neutrino particle detection data down-sampled and adjusted to 32×32 pixels.

The neutrino scattering dataset was collected at Fermi National Accelerator Laboratory as part of the MINERvA experiment that is focused on vertex reconstruction [17]. In the Main Injector Experiment for v-A (MINERvA) experiment, many scintillator strips were arranged in planes orthogonal to the neutrino beam within the detector aligned across three different orientations or “views”. We utilized both the energy lattice and the time lattice information in the dataset. In particular, we represented the energy lattice as an image, where the intensity of each pixel in the image corresponds to the average energy over time in the detection event. The images show the trajectory of particles over time from the view of one particular plane. We also used the time lattice in one of our experiments. For the time lattice, each data point in a detection event corresponds to the time at which every level exceeds a certain threshold. Associated with each detection event is a number corresponding to a specific detection plate within the chamber; this number indicates which plate a neutrino strikes. This number can then be utilized to determine in which detector region or segment the vertex of the event was located.

In BM experiments we used down-sampled and collated image data from one single plane. We did not use the original data because the quantum annealer has limited space for storing problems and because BMs are not well-suited to handling temporal data. However, the SNN experiments did take advantage of temporal data because SNNs are designed to handle such data. We offer more explanation on SNNs in Section 1.3.

Consideration of which deep learning networks to study on these platforms came next. Initially, CNNs seemed like an appropriate option, especially for HPC, but we ran into problems when considering the quantum environment. CNNs had consistently provided superior performance on standard datasets and had proven quite popular in the deep learning community. On a quantum platform, however, it became unclear how to effectively implement a CNN. Neither the circuit nor adiabatic optimization models offered good fits for CNNs, which operate using many successive layers of units. On the other hand, BMs and their probabilistic units were more like the sort of optimization problem that D-Wave hardware solves. Additionally, the quantum architecture allowed for intralayer connections between units that would normally be intractable for conventional machines to compute. Meanwhile, neuromorphic hardware running SNNs provided native time-based analysis models. BMs running on D-Wave and SNNs running on neuromorphic hardware were potentially offering distinct capabilities we believed could augment or strengthen CNN models trained in an HPC environment.

With this in mind we hope the following sections will illustrate the benefits of these different platforms. First we describe how we used a quantum annealer to train a BM containing intralayer connections and utilized the hardware to approximate samples from more complex probability distributions. Then, we show how we used a high performance computing cluster to automatically discover near-optimal topologies and parameters with evolutionary algorithms. Finally, we discuss how we natively implemented trained models produced by the previous two platforms on memristive hardware running spiking neural networks.

Because the adiabatic quantum computation portion of this project is of particular interest for this article, we next provide a more detailed description of the process and of the annealing hardware. Descriptions of the corresponding HPC and neuromorphic portions are left to Appendix B.1 and Appendix B.2.

### 3.1. Adiabatic Quantum Computation

Adiabatic quantum computation (AQC) is an implementation of the ideas of quantum computing that relies on the adiabatic theorem of quantum mechanics. This result states that if a system is in the ground state of a particular Hamiltonian and the parameters of this Hamiltonian are changed slowly enough, the system will remain in the ground state of the time-dependent Hamiltonian. This idea was used by Farhi et al. [13] to propose an alternative to the quantum circuit model of quantum computing. The main idea is to start with a Hamiltonian whose ground state is easy to construct, and slowly change it into one whose ground state encodes the answer to a particular problem.

One application of AQC is to solve combinatorial optimization problems, a particular example of which is finding the ground state of an Ising model. This model describes a system of interacting magnetic moments subject to local biases. This problem was shown by Barahona [18] to be NP-hard, so many other optimization problems of practical interest can be recast in this form. If we consider a set of spin variables $S_{i}=\pm 1$, the energy of the system is given by a quadratic expression of the form

(1) $$E_{Ising}\left(s\right)=\sum_{i}h_{i}\;s_{i}+\sum_{i,j}J_{ij}s_{i}s_{j}$$

Solving this problem means finding a spin configuration that minimizes this energy function. In a quantum approach, we consider a quantum system of interacting spins described by the Ising Hamiltonian

(2) $$H_{Ising}=\sum_{i}h_{i}\;\sigma_{i}^{z}+\sum_{i,j}J_{ij}\sigma_{i}^{z}⊗\sigma_{j}^{z}$$

where $h_{i}$ represent local magnetic fields and $J_{ij}$ are couplings between spin pairs. This Hamiltonian is diagonal in the $\sigma^{z}$ basis, and its ground state can be used to construct the corresponding configuration that minimizes the Ising energy above.

To solve this problem in the context of AQC we can choose an initial Hamiltonian of the form

(3) $$H_{0}=-\sum_{i}\sigma_{i}^{x}$$

that represents the effects of a transverse field applied to all spins. The ground state of $H_{0}$ consists in all spins being in the $|+\rangle =(|0\rangle +|1\rangle )/\sqrt{2}$ state. If we consider the spins as little magnetic moments, this corresponds to all spins pointing in the *x* direction. Quantum mechanically this state is separable, easy to construct (just apply a strong magnetic field in the *x* direction), and when expressed in the computational basis it is an equal superposition of all possible states.

The computation is performed by slowly changing the relative weights of $H_{0}$ and $H_{Ising}$ during the interval [0,T]

(4) $$H\left(t\right)=\left(1-\left(t/T\right)\right)H_{0}+\left(t/T\right)H_{Ising}.$$

This process is known as *quantum annealing*. The change must be slow compared to the time scale associated with the minimum energy gap of the time-dependent Hamiltonian, where we define the gap as the energy difference between the energies of the first excited state and the ground state [19,20,21]. If the change is too fast the system can transition to an excited state, and the state at the end of the annealing will not be the ground state of the Ising Hamiltonian. On the other hand, if the change is too slow the computation will take a long time. The main challenges in adiabatic quantum computing are to understand the connection between this energy gap (i.e., the runtime) and the size of the problem, and to find Hamiltonians that solve a given problem while possessing a larger gap [22]. However, other issues are also important for practical implementations, in particular how unavoidable noise affects the system due to the system’s interaction with the environment.

### 3.2. The Superconducting Quantum Adiabatic Processor

**Figure 4:**
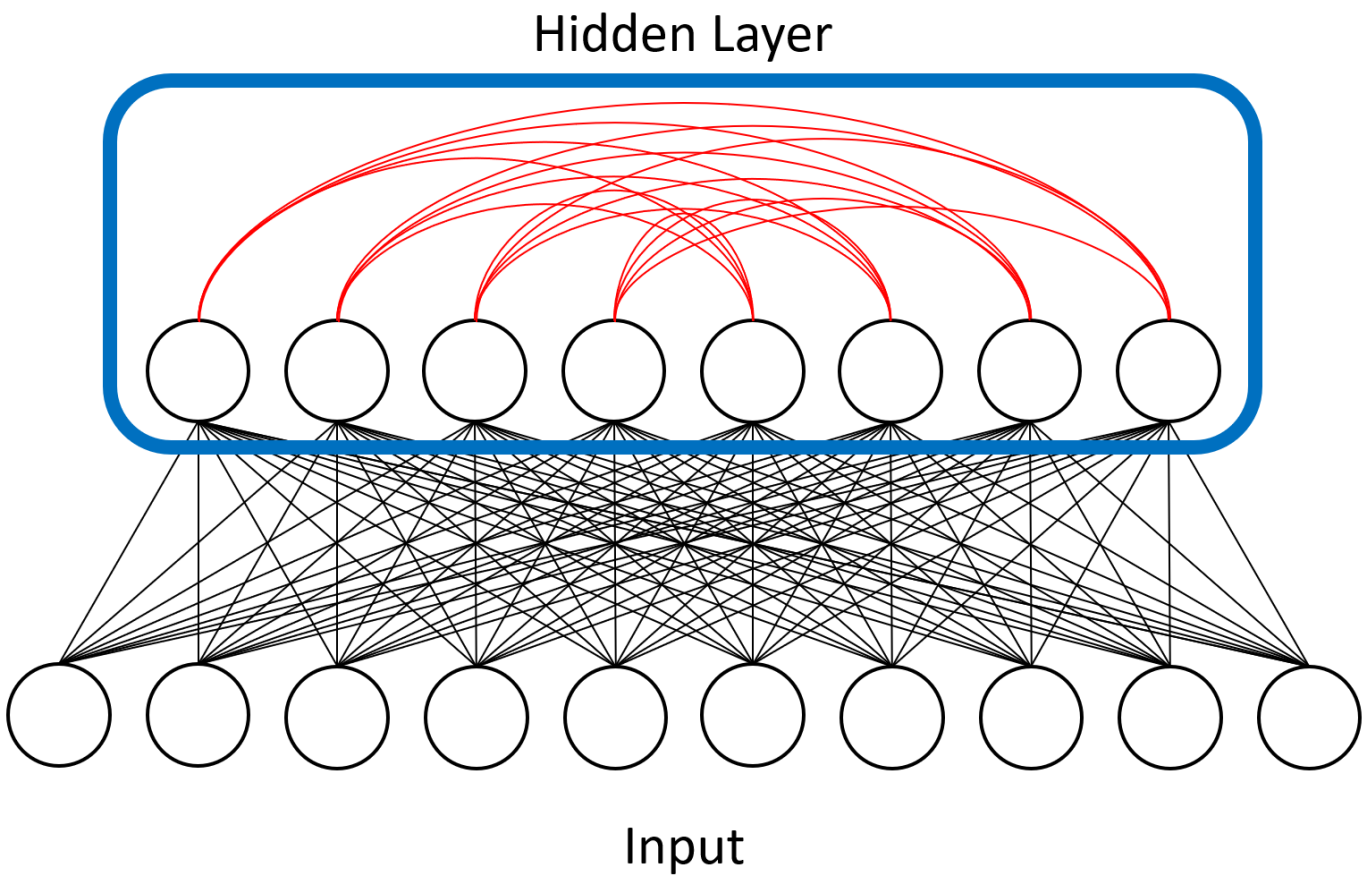
Our LBM model added connectivity between units in the hidden layer, shown in red. RBMs prohibit such intralayer connections because they add too much computational complexity for classical machines. We represented the hidden layer (outlined in blue) on the D-Wave device. The connections between hidden units were 4-by-4 bipartite due to the device’s physical topology constraints.

The architecture and physical details of the quantum adiabatic processor we studied are described in detail in [23]. In essence, it is designed to represent the Ising Hamiltonian as an array of superconducting flux qubits with programmable interactions. The qubits are implemented using superconducting quantum interference devices (SQUIDs) composed of a Niobium loop elongated in one direction. Several loops and Josephson junctions are added to the design to both allow for the required controls to implement quantum annealing and to compensate for the slight differences between the physical properties of any two SQUIDs due to fabrication variations. The processor has a unit-cell structure composed of 8 qubits with four arranged horizontally and four vertically such that each vertical qubit intersects every horizontal one. At these intersections another SQUID is placed to control the magnetic coupling between the corresponding horizontal and vertical qubits. These are the only couplings allowed (i.e., horizontal qubits are not coupled to other horizontal qubits). This architecture results in a coupling graph that is fully bipartite at the unit cell level. The processor is then built by adjoining more unit cells in a square lattice such that the horizontal qubits in one cell are coupled to the horizontal qubits in the neighboring cells to the right and the left, and the vertical qubits are coupled to the vertical qubits on top and on the bottom. A visualization of this setup, also known as a Chimera graph, is shown in Figure 4.

Programmable interactions and biases are used to implement the Ising Hamiltonian in Equation (2). The parameters $h_{i}$ represent local magnetic fields while the parameters $J_{ij}$ are the couplings between two spins. Their values are restricted to the range [−2,2] for the local local fields, and [−1,1] for the couplings. It is understood that the couplings $J_{ij}$ are only nonzero when there is a physical coupler associated with that particular pair of qubits on the chip. A transverse field term can also be implemented on each qubit, resulting in a driver Hamiltonian of the form shown in Equation (3). The adiabatic quantum computation is implemented by combining the two Hamiltonians above and changing their relative weight adiabatically, such that the system remains always in the ground state. In other words, the processor implements the Hamiltonian

(5) $$H\left(t\right)=A\left(t\right)H_{x}+B\left(t\right)H_{Ising}$$

where the functions *A* and *B* satisfy A(0)>>B(0) and A(T)<<B(T), for some final annealing time *T*. At t=0, the system is in the ground state of the transverse field Hamiltonian $H_{x}$, corresponding to all the qubits being in the same eigenstate of $\sigma^{x}$, or in other words, a superposition of all possible states in the computational basis. For the closed system case (where there are no interactions with the environment), if the quantum annealing is done slowly enough, the adiabatic theorem of quantum mechanics guarantees that the state of the system at time *T* is with high probability the ground state of $H_{Ising}$. How slow is “slowly enough” depends on the details of the Hamiltonian, in particular the inverse of the energy gap between the ground state and the first excited state, and this feature is the main factor in determining a lower bound on the run time of the device. However, real devices are not ideal closed systems, so unwanted interactions with the environment will try to kick the system out of its ground state.

The current generations of D-Wave machines are designed for experimental use and are not optimized for turnaround time, unlike relatively mature CPU or GPU platforms. Rather than directly competing against existing classical solutions to machine learning, we focus on showing it is viable to use a quantum annealer to help train a neural network with complex topologies using architectures and approximations that differ from what has been used before [14,15,16]. For this reason, instead of using clock timings, we measure error metrics against the number of training epochs. As quantum annealing technology becomes more developed, machine learning algorithms may see benefits from using this new type of hardware. Regardless, clock timings are still important to consider. We next describe the computational workflow for each problem using D-Wave machines and communication latency between a client machine and a D-Wave machine; later we describe the timings over various operations on the hardware.

Each problem is sent across a network using D-Wave’s Solver API (Matlab or Python) to the worker queue. Workers can concurrently process multiple requests and submit post-processed requests to the quantum processing unit (QPU) queue. Each request is then run sequentially on the QPU. Finally, the workers return the results back to the client. In one study D-Wave reported the mean turnaround time for each request was approximately 340 ms. Timings can vary depending on network latency-request latency can be reduced by placing the client physically next to the annealer, for example.

Communication latency aside, we also look at how long it takes to define and solve a problem on D-Wave. Loading and defining a problem on D-Wave hardware takes around $t_{d}=10$ ms. Drawing a sample from the defined distribution via annealing takes around $t_{a}$ = 20 μs. Reading out the unit states from a sample takes around $t_{r}$ = 120 μs. We repeat the sampling and read-out stages k=100 times for each MNIST image or neutrino detection instance in our experiments. So for each data point within our datasets, it takes $T=t_{d}+k\left(t_{a}+t_{r}\right)$ time to process. Currently the problem definition time $t_{d}$ and read-out time $t_{r}$ dominate wall-clock timing, but we again stress that we are looking to future developments and advancements in quantum annealing hardware that will reduce such overhead. We find the low annealing time particularly appealing because it scales well in algorithmic terms. That is, we can add additional hardware qubits or connectivity to produce more complex networks but the sampling time (annealing time $t_{a}$ for our experiments) will not increase, which is not the case for simulating equivalent networks in software.

The number of physical couplers restricts the set of problems that can be natively implemented on the processor, and it represents one of the main limitations of the devices. Minor graph embeddings can overcome this limitation but at the expense of utilizing more than one qubit per graph node [24]. As we will show in the next section, our approach turns this problem on its head. Instead of trying to fit a problem into a particular topology, we start with our hardware topology using RBMs that have no intralayer couplings and study the advantages gained from adding additional couplers.

### 3.3. Implementing a Boltzmann Machine on D-Wave

We used D-Wave’s adiabatic quantum computer located at the University of Southern California Lockheed Martin Quantum Computing Center. We implemented a Boltzmann machine to represent the MNIST digit recognition problem and neutrino particle detection problem. Deep learning using BMs has been proposed before, but as discussed in Section 2, learning is intractable for fully connected topologies because we need to compute expected values over an exponentially large state space [1,25]. RBMs address this by restricting network topology to bipartite connectivity to introduce conditional independence among “visible” units (representing the dataset and RBM output) given the “hidden” units (representing latent factors that control the data distribution), and vice versa, though they lose some representational power in the process. The quantum annealing hardware gave us an opportunity to first implement an RBM to establish baseline performance and then ease some topology restraints to investigate how more complex topologies could improve our results.

Our RBM used 784 visible units to represent each pixel in a 28×28 MNIST digit image and 80 hidden units on a D-Wave adiabatic quantum computer. We added an additional 10 visible units as a digit classification layer where the unit with highest probability was chosen as the label. Similarly we used 32×32=1024 units to represent the neutrino data, 80 hidden units, and 11 classification units to represent the 11 collision sites in the neutrino detection chamber, where the classification unit with the highest probability was chosen as the BM’s guess for which plate the particle struck. The BMs were trained over 25 epochs on a training set and then evaluated against a validation set.

Next, as mentioned above, we loosened some of the topology restrictions of RBMs. RBMs enforce bipartite connectivity (see Figure 1), meaning hidden units are not connected to one another. We partially removed this restriction and allowed some of our hidden units to communicate with each other. We called this semi-restricted BM a “limited” Boltzmann machine (LBM). LBMs can be viewed as a superset of RBMs, the only difference being a set of extra available connections between hidden units. The previously described superconducting quantum adiabatic processor has physical constraints that limit connectivity to a chimera topology, so LBMs remain a subset of BMs.

**Figure 5:**
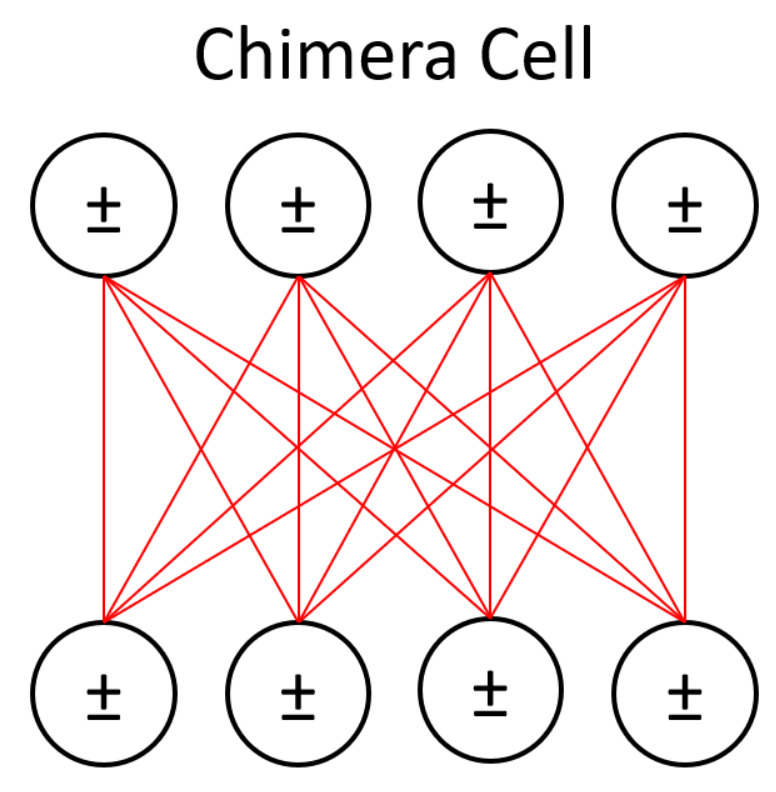
The hidden layer from Figure 4 is represented in one of D-Wave’s chimera cells here, with the cell’s bipartite connectivity made more obvious. The input/visible units of the LBM are left on a classical machine. Their contributions to the activity of the hidden units is reduced to an activity bias (represented with ± symbols) on those units. Figure 6 shows the overall chimera topology of the D-Wave device.

Because D-Wave hardware faces a physical constraint on the number of possible units and connections, we would have had to employ the minor embedding approach mentioned above if we wanted to represent all of a BMs units on hardware. This would result in a large overhead in the number of qubits required, restricting our approach to small BMs. However, we can still try to exploit the quantum features of the D-Wave by restricting the topology of our model and only embedding part of it in the device. In our implementation we chose to represent only the hidden units, used the annealer as a sampler for the interconnected hidden units to estimate required quantities needed to update the weights, and left representation of the visible units to a classical machine. We were primarily interested in the interaction between hidden/latent units because they can represent abstract features extracted from the data. Figure 4 visualizes the extra connectivity we added to the LBM model and Figure 5 shows how we represented LBMs on the D-Wave’s chimera topology.

Using D-Wave hardware to adjust LBM parameters may help tackle the intractability issue because the quantum annealer does not rely on conditional independence between units within a layer. We give a short explanation of the training process for BMs to illustrate.

The configuration *x* of binary states *s* of units has an energy *E* defined by

(6) $$E\left(x\right)=-\sum_{i}s_{i}b_{i}-\sum_{i<j}s_{i}s_{j}w_{ij}$$

where *b* is the bias of a unit and $w_{ij}$ is the mutual weight between two units *i* and *j*. The partition function is $\sum_{u}e^{-E(u)}$, and the probability the BM produces a particular configuration *x* is

(7) $$P\left(x\right)=e^{-E(x)}/\sum_{u}e^{-E(u)}.$$

P(x) is difficult to compute in a full BM because it requires a sum over an exponentially large state space. If we want to determine the probability of some hidden unit *i* is on (equal to 1) without any guarantee of conditional independence, we would have to calculate $P\left(h_{i}=1\right)=P\left(h_{i}=1|v,h_{-i}\right)$ where *v* is the state configuration of visible units and *h* is state configuration of the hidden units. However, if we use RBMs to restrict ourselves to bipartite connectivity between *v* and *h*, this probability factorizes and we can write $P\left(h_{i}=1\right)=\prod_{j=1}^{n}P\left(h_{i}=1|v_{j}\right)$. Our first RBM baseline experiment used this standard procedure with 1-step Gibbs sampling. In our LBM experiment, we did not need to rely on conditional independence or Gibbs sampling because we used quantum annealing instead to approximate samples from the more complicated probability distribution.

The training procedure for BMs compares the distribution of the data against the expected distribution according to the model and uses the difference to adjust the weight matrix *w*. Sampling from the model is difficult so we approximate using Markov Chain Monte Carlo (MCMC) sampling. The first “positive” phase of training locks the states of visible units to a configuration determined by the data—for example, a 28×28 pixel image from the MNIST dataset. The hidden unit distribution according to the data is found in this phase. The second “negative” phase unlocks the visible units and the system is allowed to settle. Sampling during this phase is difficult so we approximate samples using contrastive divergence with one step of MCMC and find the unit distributions according to the BM model. The weight matrix is then updated with the following equation:

(8) $$\Delta w_{ij}=\epsilon \left({\langle v_{i}h_{j}\rangle }_{data}-{\langle v_{i}h_{j}\rangle }_{reconstruction}\right)$$

where ϵ is the learning rate, ${\langle v_{i}h_{j}\rangle }_{data}$ is the product of visible and hidden unit state probabilities in the positive phase, and ${\langle v_{i}h_{j}\rangle }_{reconstruction}$ is the product of visible and hidden unit probabilities in the negative phase.

For the MNIST problem we used 6000 images from the MNIST digit dataset to train the RBM and LBM. Each 28×28 image was represented with a 784-length vector with 10 units using 1-hot encoding to represent the class of digit. In training the labels were hidden and the BM attempted to reconstruct them to guess what the image label was. The classification unit with the highest probability of being “on” was chosen as the BM’s label guess. The neutrino experiment used the same setup except the images were 32×32 pixels and thus there were 1024 visible units. The weight matrices were randomly initialized from a standard normal distribution and updated using the rule in Equation (8).

We wanted to further explore how connections between hidden units, referred to as couplers, contributed to problem solving in an LBM topology. To do so we limited the visible-to-hidden connectivity in the next experiment such that each hidden unit was only allowed to see a 4×4 box of pixels in the input images. These boxes did not overlap with each other. Reconstructing the input image became a much harder problem and the hope was that the addition of couplers would allow hidden units to trade information about input pixels in boxes they normally could not communicate with and improve results. This setup was somewhat inspired by CNN convolutional layers but we decided to make the “convolution” non-overlapping to use fewer qubits. In the future we will expand to use more qubits.

We believed this setup would make couplers relatively more important to the LBM because we reduced the ratio of visible-hidden connections to couplers. An input image with $32\times 32=1024=2^{10}$ data points and 64 hidden units has $2^{10}\times 2^{6}=2^{16}$ visible-to-hidden connections for 168 couplers. However, hidden units with only 4×4 boxes of pixel visibility would instead have $2^{4}\times 2^{6}=2^{10}$ visible-to-hidden connections for 168 couplers.

## 4. Results

**Figure 7:**
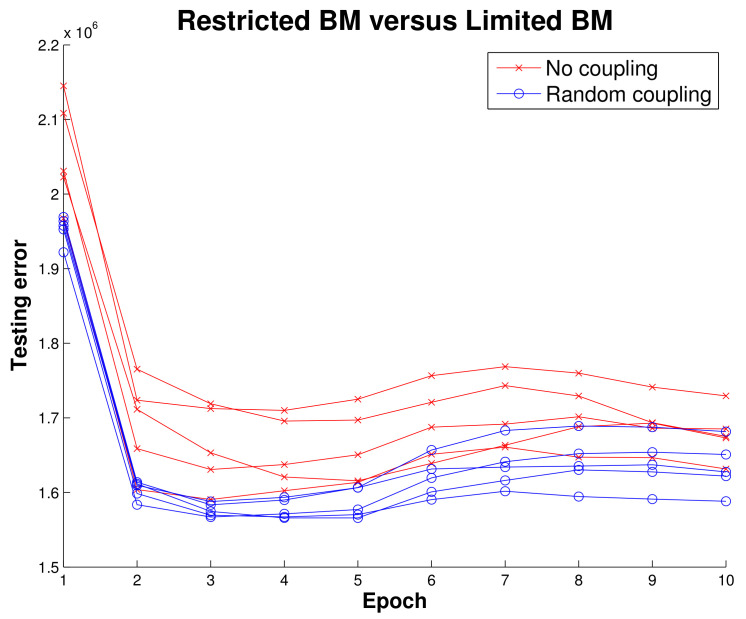
An initial experiment to demonstrate LBM utility. Reconstruction error (sum of squared error) of BMs trained on simulated data using no intralayer connections and using random intralayer connections with a small (0.0001) hidden-to-hidden weight learning rate. Here we show five RBMs (**red**) and five LBMs (**blue**), and the results suggest even just the presence of relatively static intralayer connections gives LBMs a performance advantage over RBMs. We obtained these results from the quantum annealing simulator provided by D-Wave.

We trained our RBM and LBM using the same parameters over 25 epochs (complete runs over all the training data). We followed common guidelines for choosing and adjusting hyperparameters [26]. We selected the learning rate ϵ to be 0.1 for weights between visible-to-hidden weights and 0.1 for hidden-to-hidden units for our experiments, excepting our first one shown in Figure 7. Setting ϵ too low means a BM learns slowly and may get trapped in local minima whereas setting it too high can cause the network to travel wildly in parameter space and be unable to learn coherently.

Before implementing the RBM running on MNIST data we wanted to get initial results indicating there was some merit to the LBM topology. Using simulated data, we mapped a BM to a quantum annealing simulator and trained two configurations, one where intralayer connections were disabled and one that had random intralayer connections. Ten epochs of training an RBM and LBM in Figure 7 show that LBM has some advantage.

**Figure 8:**
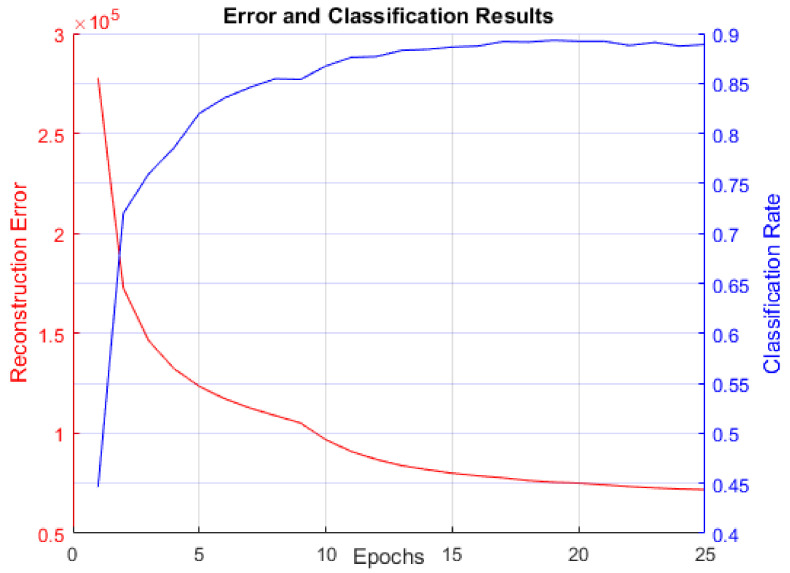
Reconstruction error and classification rate over 25 training epochs using 6000 MNIST images for training and 6000 for testing. Reconstruction error decreases as classification rate rises, confirming that the RBM learns the MNIST data distribution.

**Figure 9:**
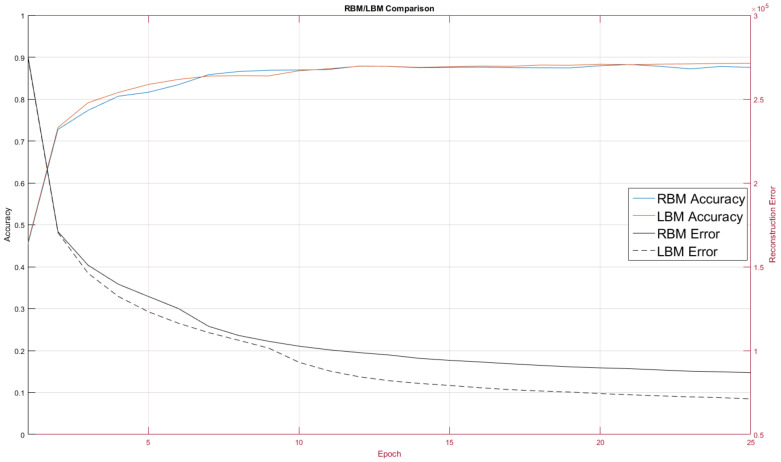
RBM and LBM performance on the MNIST digit classification task. The LBM tends to label the digits slightly better and produces lower reconstruction error than the RBM.

As discussed, our first experiment was to establish performance baselines in RBMs so we could later compare LBMs against them. Figure 8 displays reconstruction error (sum of squared error between the actual data and BM reconstruction data mentioned in Section 3.3) and classification rate. This figure is included to confirm that the RBM did indeed learn to model the MNIST digit data distribution. Figure 9 contains a comparison of RBM performance and LBM performance on the MNIST digit recognition problem.

The RBM and LBM were both implemented on D-Wave and on MNIST images using the same number of hidden and visible units. For this test we trained over 10 epochs. The RBM configuration, as discussed, had no intra-layer connections, whereas the LBM configuration had limited connections between the hidden nodes.

One quirk we found was LBM configuration initially performed worse than the RBM configuration. This was unexpected and we adopted a hybrid learning approach where the intralayer connections were reassigned from a random normal distribution for the first three training epochs. Afterwards the intralayer couplers were allowed to evolve according to the standard training rule. Our choice of a 3-epoch delay for intralayer training was rather arbitrary; further exploration into the mechanics involved will be explored in future work where we will pre-train models as RBMs on classical machines and then later hand over training to a quantum annealer.

The LBM achieved a classification rate of 88.53 percent, seen in Figure 7, and was comparable to other RBM results on MNIST [27].

Our LBM setup mapped only the hidden units to the D-Wave hardware whereas most other works map a whole BM. The latter approach requires down-sampling and graph embedding. We hope our approach scales better with problem size because we represent the visible input units on classical machines and still use contrastive divergence as a training method.

Our experiments on neutrino data and limited visible-to-hidden connectivity were run on both simulation software and D-Wave hardware. We used both because hardware has physical limits regarding parameter ranges and experiences parameter warping, so the inclusion of software results provides additional support if both environments produce comparable results. Parameters on the hardware for Ising models have around 4–5 bit precision and can only take on values within a small range, typically [−2,2] for *h* or [−1,1] for *J*. Software simulators do not have this limited precision and their parameters are not limited to any particular range.

**Figure 10:**
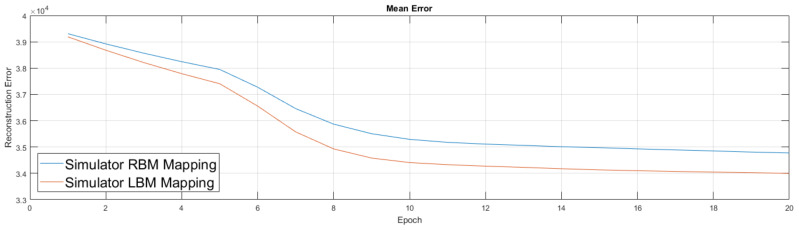
Comparison of RBM against LBM trained on neutrino data using a software simulator. Weights are randomly initialized from a normal distribution. The change in learning rate at epoch 5 is due to a change in the momentum parameter in the algorithm that is designed to speed the rate of training. The graph shows the mean performance of five different RBMs and five different LBMs and suggests the mean reconstruction error of RBM and LBM are significantly different.

**Figure 11:**
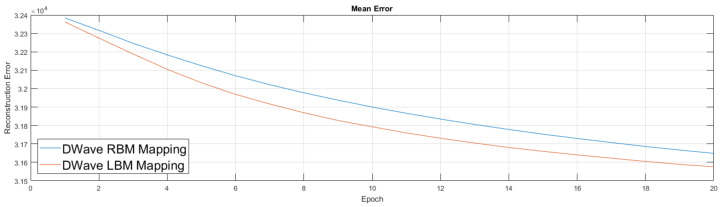
Another comparison of RBM against LBM run on neutrino data using D-Wave hardware. Both the RBM and LBM are initialized from the same pre-trained model. The pre-trained model is an RBM run for three epochs on a classical machine. The graph shows the mean performance of five different RBMs and five different LBMs, suggesting the performance difference between RBM and LBM persists on hardware.

We show the simulator results in Figure 10. Results from the simulator suggest the addition of couplers in this new setup improved performance, which led to our move to experiment on the quantum annealing hardware. Our experiments in Figure 11 were similar to the previous ones, albeit we first trained an RBM on a classical machine. We then took this lightly trained RBM model and moved it to the D-Wave hardware, used its semi-trained parameters to initialize the weights of the D-Wave RBM and LBM, enabled 168 couplers, then continued training for an additional 20 epochs. We again performed the RBM experiment five times and the LBM experiment five times.

In the LBM experiment we did not remap qubits in any scheme more complicated that a linear fashion. That is, we designated each qubit to oversee a 4×4 box in a horizontal order and simply assigned each qubit to unit cells according to this order. In future work we will argue this is suboptimal and that we can improve our results even more by considering smarter remappings of qubits to take advantage of locality within image data. For now we leave the comparison as RBM results versus LBM results without any special qubit remapping.

One aspect of superconducting technology worth mentioning is power consumption. The energy consumption of a system such as the D-Wave hardware is dominated by the cooling of the processor. When programming the device, the control signals inject some energy into the system that can increase the temperature by a few million Kelvin. This energy needs to extracted, resulting in a few pico Watts of power being dissipated in this step. However, the actual computation requires a negligible amount of energy. The cooling requirement has remained flat for four generations of the D-Wave device and is not expected to change in the foreseeable future. While the energy consumption of quantum annealers is typically not a highlighted advantage over classical systems, power efficiency may eventually become an important reason for preferring quantum computing systems in the future.

## 5. Alternative Approaches

We have mentioned HPC and neuromorphic technology as two other platforms that can be utilized to benefit deep learning networks. Each has certain qualities that are not found in our adiabatic quantum computation approach due to fundamental differences between the platforms. Quantum annealers can handle complex topology but are limited in number; HPC exploits massive parallelization for computation speed but still uses classical machines; neuromorphic hardware is low power but tricky to train. We envision an integrated future where we can call upon the strengths of each platform to augment machine learning efforts. In this section we describe results from our HPC and neuromorphic efforts and how they can also contribute to training deep learning networks.

### 5.1. HPC

In previously reported work [28] we demonstrated that improved network hyperparameters can be found by using an evolutionary algorithm [29] and the Titan supercomputer, a collection of 300,000 cores and 18,000 Nvidia Tesla K20x GPUs. These results demonstrated that near optimal hyperparameters for CNN architectures can be found for the MNIST handwritten digit dataset by combining evolutionary algorithms and high performance computing. The kernel size and the number of hidden units per layer were the hyperparameters that were optimized. This work utilized 500 nodes of Titan for 3 h in order to evaluate 16,000 hyperparameter sets.

An improved version of the aforementioned evolutionary algorithm has been developed such that not only can hyperparameters of a fixed topology be optimized, but the topology of the network itself can be optimized [30]. This improved algorithm can evolve the number of layers and the type of each layer in addition to each individual layer’s hyperparameters. This work has been applied to the MINERvA vertex reconstruction problem, which we have referred to as the neutrino particle detection problem in this paper, and has yielded improved results over standard networks. This approach is able to achieve an accuracy of 82.11% after evaluating nearly 500,000 networks on Titan in under 24 h utilizing 18,000 nodes of Titan, which represents a significant improvement over the baseline network that achieved 77.88%. Manually designing a network to attain such an improvement could take weeks or months due to the limited ability of a human to design, evaluate, and interrogate the performance of their networks in order to propose improved designs.

These HPC results are relevant to our quantum annealing approach because efforts to apply AQC to deep learning networks can benefit from this ability to pick good hyperparameters. When we designed our RBM and LBM experiments, we manually chose learning rates and topologies. Future work can incorporate our HPC findings here to find optimal hyperparameters for our deep learning networks before using a quantum annealer to further tune the networks. Our LBM experiment where we first trained an RBM on a classical machine before moving it to the annealer and adding intralyer connections seems particularly amenable to such a procedure.

### 5.2. Neuromorphic

The neuromorphic approach fits into the context of our overall project through its potential for low-power implementations of networks derived from the AQC and HPC portions of our work. AQC needs hardware to be cooled as much as possible and HPC needs thousands of CPUs/GPUs. The power consumption of either is far beyond what a neuromorphic solution requires to function.

For our neuromorphic comparison points we considered a two-phase experiment. The initial phase was to demonstrate the feasibility of a native spiking neuromorphic solution by implementing an SNN in a software-based simulation. The next phase was to collect energy estimates by simulating the characteristics of the corresponding SNN implemented on memristive neuromorphic hardware. In a previous work [28] for the MNIST task, we started by simulating a simple spiking neural network trained to classify MNIST images.

We used evolutionary optimization (EO) to generate an ensemble of networks that classified MNIST images with an accuracy of approximately 90%. The accuracy of the generated ensemble was comparable to some other non-convolutional spiking neural network approaches [27]. The network we considered for this experiment was one network in the ensemble. In particular, the network we chose is one that distinguishes between images of the digit 0 and images of other digit types. For the second phase of the experiment the energy consumption was also determined for a memristive implementation of this network. Here the synapses consisted of metal-oxide memristors and represented both a weight value and a delay value. Each synapse in the network had twin memristors to implement both positive and negative weights [31] and a synaptic buffer to control the delays and peripheral connections. The neurons used in the network are implemented using the mixed-signal integrate and fire approach.

The simulation of energy estimate leveraged the energy per spike values for each synapse and neuron phases gathered from low-level circuit simulation. The network was simulated with a clock speed of 16.67 MHz and the average power and energy calculated for the network was 304.3 mW and 18.26 nJ. We note that this estimate includes the digital programmable delays as well. However, if we consider the core analog neuromorphic logic, the energy per spike is 5.24 nJ and the average power was 87.43 mW, which is consistent with similar memristor-based neuromorphic systems [32]. In contrast, MNIST classification tasks on GPU, field-programmable gate arrays (FPGA), or even application-specific integrated circuit (ASIC) architectures were reported to be in the W range [33], far above neuromorphic implementations like the one we described or IBM’s TrueNorth [34].

In previous work [35] we also applied this approach to estimating the energy usage of a memristive based implementation on the Fermi data. As opposed to the MNIST task in which we trained multiple SNNs to form an ensemble, we built a single SNN for the neutrino data with 50 input neurons and 11 output neurons where the 11 output neurons corresponded to the 11 class labels in the neutrino data. We used a single view of the data (the x-view) rather than all three views. Instead of interpreting the data as pixels in an image we utilized the time lattice of the data. In the time lattice each value in the x-view corresponds to the time at which the energy at that point exceeded a low threshold. We used these times to govern when spikes should appear as input in the SNN. This generated a natural encoding for SNN-style networks as opposed to the somewhat unnatural mapping of non-temporal data to an image format. We found a resulting network with 90 neurons and 86 synapses that reached approximately 80.63% accuracy on the testing set, comparable to the approximately 80.42% accuracy achieved by a CNN that was also restricted to the x-view [17]. We estimated the energy usage of a memristive based neuromorphic implementation of the network for the neutrino data to be approximately 1.66 μJ per classification. These results, more so than the MNIST results, demonstrate that leveraging the temporal nature of certain data may result in extremely efficient SNN solutions to certain tasks.

## 6. Discussion

**Figure 12:**
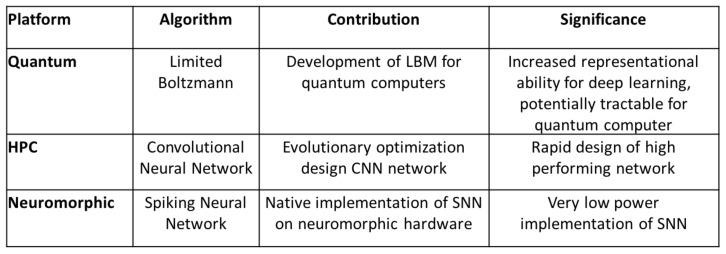
A comparison of the platforms, deep learning approaches, contributions, and significance of the result from the MNIST experiment.

We compared a standard benchmark problem, MNIST digit recognition, on three different platforms: quantum adiabatic optimization, HPC, and neuromorphic. Our results show each option offers a unique benefit. Quantum adiabatic computation opens up complex topologies for use in deep learning models that would normally prove intractable for classical machines. HPC allows us to optimize CNNs on a large scale to find an optimal topology with its associated parameters. Neuromorphic lets us implement low power neural network solutions derived from other platforms. Figure 12 provides a summary of these platforms and their associated qualities. However, it is also clear that the MNIST problem is not ideally suited to showcase the capabilities of either the quantum or neuromorphic systems because it has been essentially solved using CNNs.

**Figure 13:**
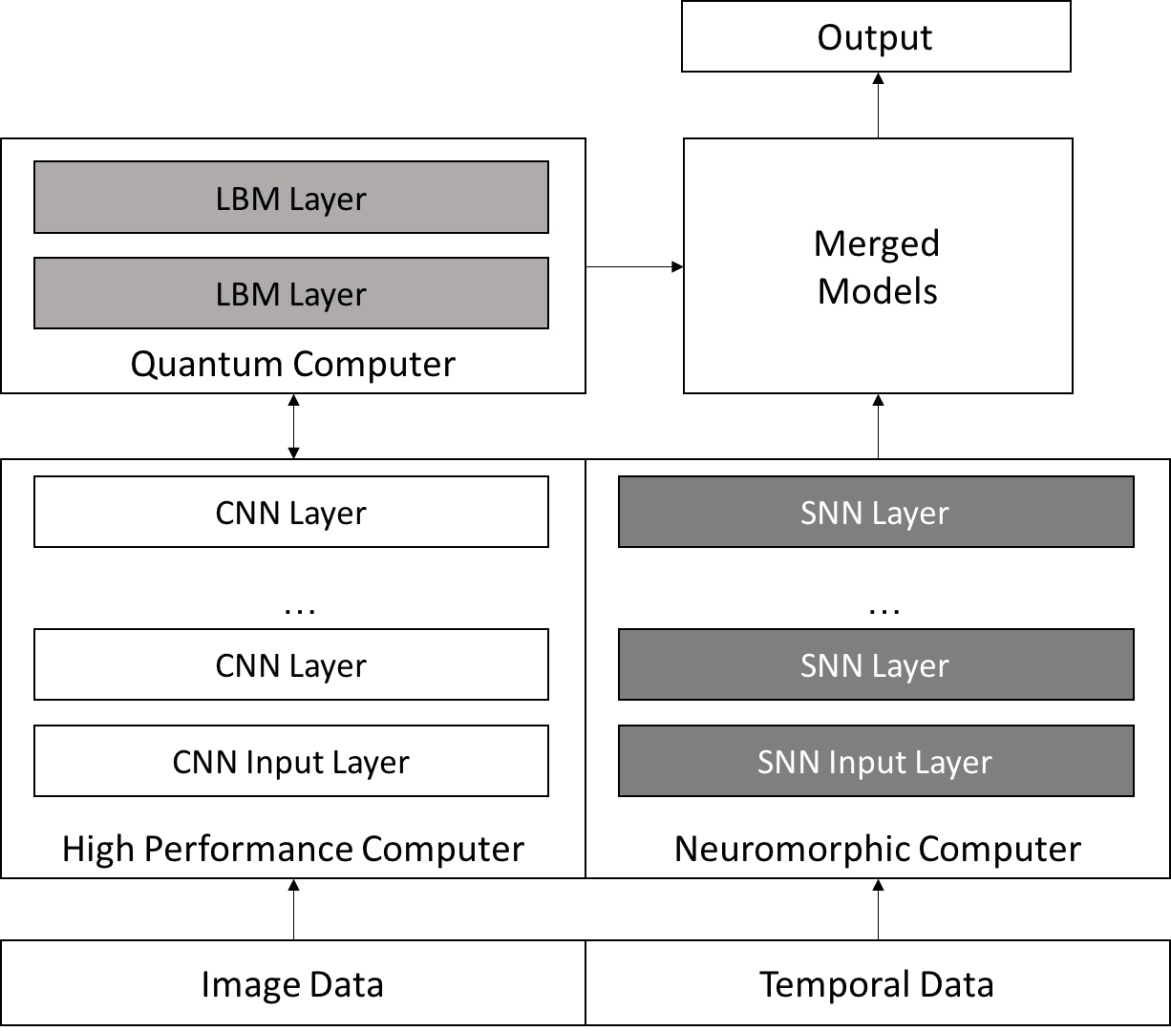
A proposed architecture that shows howthe three approaches, quantum, HPC, and neuromorphic can be used to improve a deep learning approach. Image data can be analyzed using an HPC rapidly derived CNN with the top layers using an LBM on a quantum computer. The top layers have fewer inputs, and require greater representational capabilities which both play to the strength and limitations of a quantum approach. The temporal aspect of the data can be analyzed using an SNN. Finally, the image and temporal models will be merged to provide a richer and we believe a more accurate model, with an aim to be deployed in very low power neuromorphic hardware.

For example, the greater representational power of the quantum LBM approach is likely better utilized on a more complex dataset. Similarly, spiking neuromorphic systems may be better suited for use on datasets that include temporal components. In Figure 13 we propose an architecture we believe provides the ability to leverage the strengths of each of these computing platforms for future, more complex data sets.

The goal of this study is to explore how to address some of the current limitations of deep learning, namely networks containing intralayer connections, automatically configuring the hyperparameters of a network, and natively implementing a deep learning model using energy efficient neuron and synapse hardware. We used quantum computing, high performance computing, and neuromorphic computing to address these issues using three different deep learning models (LBM, CNN, and SNN).

The quantum adiabatic computing approach allows deep learning network topologies to be much more complex than what is feasible with conventional von Neumann architecture computers. The results show training convergence with a high number of intralayer connections, thus opening the possibility of using much more complex topologies that can be trained on a quantum computer. There is no time-based performance penalty due to the addition of intralayer connections, though there may be a need to sample more often in order to reduce potential errors.

HPC allows us to automatically develop an optimal network topology and create a high performing network. Many popular topologies used today are developed through trial and error methods. This approach works well with standard research datasets because the research community can learn and publish the topologies that produce the highest accuracy networks for these data. However, when the dataset is relatively unknown or not well studied, the trial-and-error approach loses its effectiveness. The HPC approach provides a way to optimize the hyper-parameters of a CNN, saving significant amounts of time when working on new datasets, perhaps even bootstrapping under-studied datasets into the regular publish-and-review iterative process.

Memristor-based hardware provides an opportunity to natively implement a low-power SNN as part of a neuromorphic computing environment. Such a network has the potential to feature broader connectivity than a CNN and the ability to dynamically reconfigure itself over time. Neuromorphic computers’ benefits, including robustness, low energy usage, and small device footprint, can prove useful in a real-world environment today if we develop a mechanism for finding good network solutions for deployment on memristor-based devices that do not rely on conversions from non-spiking neural network types.

We can use the three different architectures together to create powerful deep learning systems to go beyond our current capabilities. For example, current quantum annealing hardware is limited in the size and scope of problems it can solve but does allow us to use more complex networks. We can turn this into an opportunity by using a complex network as a higher level layer in a CNN as seen in Figure 13. Higher layers typically combine rich features and can benefit from increase intralayer connectivity; they also have smaller-sized inputs than lower layers, easing the limited-scope issue of current quantum annealing hardware. Such an augmented CNN may improve overall accuracy.

The HPC approach of automatically finding optimal deep learning topologies is a fairly robust and scalable capability, though quite expensive in development and computer costs. The ability to use deep learning methods on new or under-studied datasets (such as the neutrino particle detection dataset) can provide huge time savings and analytical benefit to the scientific community.

The neuromorphic approach is limited by the lack of robust neuromorphic hardware and algorithms, but it holds the potential of analyzing complex data using temporal analysis using very low power hardware. One of the most compelling aspects of this approach is the combination of a SNN and neuromorphic hardware that can analyze the temporal aspects of data. The MNIST problem does not have a temporal component, but one can imagine a dataset that has both image and temporal aspects such as a video or our neutrino detection dataset. A CNN approach has been shown to perform well on the image side, so perhaps a SNN can provide increased accuracy by analyzing the temporal aspects as well. For example, a CNN could analyze an image to detect objects within the image and output the location and/or orientation of those objects. This output can be used as input for an SNN. As each video frame is processed independently by the CNN, the output can be fed into the SNN, which can aggregate information over time and make conclusions about what is occurring in the video or detect particular events that occur over time, all in an online fashion. In this example the CNN could be trained independently using the labeled frames of the video as input images while the SNN could be trained independently utilizing different objects with their locations and orientations as input.

These experiments provide valuable insights into deep learning by exploring the combination of three novel approaches to challenging deep learning problems. We believe that these three architectures can be combined to gain greater accuracy, flexibility, and insight into a deep learning approach. Figure 13 shows a possible configuration of the three approaches that addresses the three deep learning challenges we discussed above. The high performance computer is used to create a high performing CNN on image type data. The final layer or two is then processed by the quantum computer using an LBM network that contains greater complexity than a CNN. The temporal aspects of the data are modeled using an SNN, and the ensemble models are then merged and an output produced. Our belief is that this approach has the potential to yield greater accuracy than existing CNN models.

### Future Work

We will test the proposed architecture to determine if it provides improved accuracy, flexibility, and insight into a dataset over methods derived from a traditional CNN approach. We will apply this to neutrino particle detection data and compare the proposed architecture against other contemporary methods.

We will also investigate how qubit mapping affects LBM results. Our experiment used a simple 1:1 mapping of hidden units to qubits by placing qubits in chimera cells in the order we defined them. However, this does not take advantage of locality within data; we will examine which methods of qubit mapping produce better results and see how they reveal patterns within our datasets.

## 7. Conclusions

Though inspired by biological neural models, deep learning networks make many simplifications to their connectivity topologies to enable efficient training algorithms and parallelization on GPUs. CNNs in particular have emerged as a standard high performance architecture on tasks such as object or facial recognition. While they are powerful tools, deep learning still has several limitations. First, we are restricted to relatively simple topologies; second, a significant portion of network tuning is done by hand; and third, we are still investigating how to implement low power, complex topologies in native hardware.

We chose three different computing environments to begin to address the issues respectively: quantum adiabatic computing, high performance computing clusters, and neuromorphic hardware. Because these environments are quite different, we chose to use different deep learning models for each. This includes Boltzmann machines in the quantum environment, CNNs in the HPC environment, and SNNs in the neuromorphic environment. We chose to use the well-understood MNIST hand-written digit dataset and a neutrino particle detection dataset.

Our results suggest these different architectures have the potential to address the identified deficiencies in complex deep learning networks that are inherent to the von Neumann CPU/memory architecture that is ubiquitous in computing.

The quantum annealing experiment showed that a complex neural network, namely one with intralayer connections, can be successfully trained on the MNIST digit recognition and neutrino particle detection tasks. The ability to train complex networks is a key advantage for a quantum annealing approach and opens the possibility of training networks with greater representational power than those currently used in deep learning trained on classical machines. High performance computing clusters can use such complex networks as building blocks to compare thousands of models to find the best performing networks for a given problem. Finally, the best performing neural network and its parameters can be implemented on a complex network of memristors to produce a low-power hardware device capable of solving difficult problems. This is a capability that is not feasible with a von Neumann architecture and holds the potential to solve much more complicated problems than can currently be solved with deep learning on classical machines.

We proposed a new deep learning architecture based on the unique capabilities of the quantum annealing, high performance computing, and neuromorphic approaches presented in this paper. This new architecture addresses three major limitations we see in current deep learning methods and holds the promise of higher classification accuracy, faster network creation times, and low power, native implementation in hardware.

## Figures and Tables

**Figure 6:**
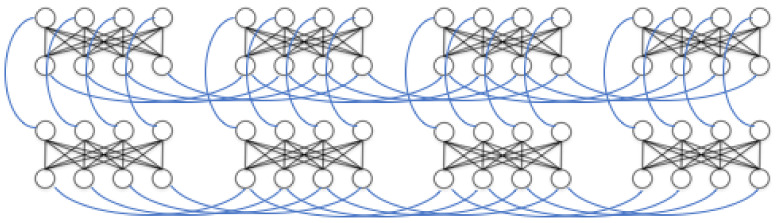
Chimera graphs are composed of 8-qubit cells featuring bipartite connectivity. Each cell’s partition is connected to another partition in the adjacent cells.

## Appendix A.

### Appendix A.1. Related Works for High Performance Computing

Deep learning, being an early adopter of GPU technology, has benefited greatly from the speedup offered by these accelerated computing devices and has received great support from device manufacturers in the form of deep learning-specific GPU libraries. General purpose GPUs are the basic building blocks of today’s HPC platforms and next generation machines will rely on them to an even greater degree. Thus, deep learning provides a great opportunity to fully utilize these machines, as they will have multiple GPUs per compute node. This leaves the question of how to best utilize thousands of GPUs for deep learning, as previous work has only utilized a maximum of 64 GPUs before encountering scaling problems when trying to exploit model parallelism to spread the weights of the network across multiple GPUs [36]. HPC provides the unique opportunity to address the problem of network specification. This refers to the problem of deciding upon the set of hyper-parameters needed to specify the network and training procedure in order to apply deep learning to a new dataset.

For convolutional neural networks, this could involve specifying parameters such as the number of layers, the number of hidden units, or the kernel size. For more general networks, such as RBMs, this could involve defining much more complicated connectivity between neurons.

Previously, it has been shown that HPC can be utilized to optimize the hyperparameters of a deep learning network [29]. This work utilized an evolutionary algorithm distributed across the nodes of Oak Ridge National Laboratory’s (ORNL’s) Titan supercomputer in order to optimize the performance of deep learning algorithms. We include the activation function used, the number of hidden units in a layer, the kernel size of a convolutional layer, and the learning rate of the solver as hyperparameters. As the size of the network grows, the hyper-parameter space grows increasingly larger. The size of deep learning networks used today have resulted in a hyper-parameter space that cannot be searched on a single machine or a small cluster. This is a result of the computational complexity of training and evaluating these networks. Without utilizing the computational capabilities provided by supercomputers, evaluating a sufficient number of hyper-parameter sets to search the enormous hyper-parameter space of these methods would be impossible.

### Appendix A.2. Related Works for Neuromorphic Computing

There are two primary reasons that researchers have pursued the development of neuromorphic computing architectures: to develop custom hardware devices to accurately simulate biological neural systems with the goal of studying biological brains and to build computationally useful architectures that are inspired by the operation of biological brains and have some of their characteristics. In developing neuromorphic computing devices for computational purposes, there have been two main approaches: building devices based on spiking neural networks (SNNs), such as IBM’s TrueNorth [37] or Darwin [38], and building devices based on traditional or convolutional neural networks, such as Google’s Tensor Processing Unit [39] or Nervana’s Nervana Engine [40], to serve as deep learning accelerators. The neuromorphic devices that have been built based on SNNs or built to simulate more biologically-accurate systems have vastly different characteristics than those that have been built based on deep learning networks, such as CNNs. The neurons in SNN-based systems are typically not organized in layers and have fewer restrictions on connectivity between neurons, allowing for more complex network topologies including recurrent networks. The neuron and synapse models also differ from those in convolutional neural networks and recurrent neural networks such as long short term memories (LSTMs) [41]. Specifically, in SNN-based neuromorphic systems, the neuron is typically some form of spiking neuron, such as a leaky-integrate-and-fire neuron, and the synapses usually have a delay value in addition to a weight value, thus introducing a temporal component to the processing of the network.

The primary computational issue associated with SNN-based systems is that few algorithms that train native networks for those systems have been developed. The key reason why algorithms have not been developed is the computational difficulty introduced by the broader connectivity in the network and the inclusion of the temporal component in both the neurons and synapses. One approach for training networks for neuromorphic computers has been to train a CNN offline and then create a mapping process from the CNN to the associated SNN-based neuromorphic hardware [42]. This mapping of an existing neural network trained with a well-studied algorithm (in this case, backpropagation) has been used for a variety of other neural network types beyond convolutional neural networks, such as spiking Hopfield networks and spiking restricted Boltzmann machines [43]. The algorithms that have been developed for spiking neuromorphic systems typically impose some sort of restriction for the network, or they have not yet been shown to be widely applicable. For example, a variation of back-propagation for spiking neural networks (SpikeProp) has been developed [44,45] but it is restricted to feed-forward networks and simply learns the weight values for the synapses. Learning rules based on spike-timing dependent plasticity or STDP have also been commonly used in spiking neural network architectures [46]. Though STDP has been shown to be useful on some tasks, including unsupervised tasks, the true impact of STDP on real applications has not yet been demonstrated. It is worth noting that STDP mechanisms have great potential to be used as unsupervised weight training method, but it may need to be used alongside a supervised algorithm that can help to determine network topology and parameters.

A key property of neuromorphic systems is their potential for more energy-efficient computation. To achieve energy-efficiency, we (and many others) have explored an implementation of a spiking neural network system utilizing memristors. Memristors are “memory resistors” in that their resistance can be altered depending on the magnitude of the voltage applied. When no voltage is applied across a memristor, the most recent resistance value is retained [47]. Memristors have similar behavior to biological synapses, and as such, have been frequently utilized to implement neuromorphic systems [48,49,50].

## Appendix B.

### Appendix B.1. Description of High Performance Computing

The high performance computer we are using is the ORNL’s Titan computer with roughly 300,000 cores, and 18,000 GPUs. This is currently the fastest open science computer in the world.

Clearly a supercomputer is not needed to solve the MNIST problem; however, a supercomputer is extremely valuable in automatically finding an optimal deep learning topology for such a problem. Rather than using a trial-and-error method for finding a well performing network topology, we utilize an evolutionary optimization on Titan to evaluate tens of thousands of topologies [29]; therefore, systematically finding the best performing networks on this problem. If achievable, this would solve one of the major challenges in building deep learning networks.

For this project we used a CNN as our deep learning network since CNNs currently produce the top results. We approached the network topology problem of selecting optimal hyper-parameters as a massive search problem, where Titan can be used to quickly search the space.

We represented each individual within the population of the evolutionary algorithm (EA) as a single deep neural network or CNN. An individual consisted of a genome where the genes represented the various hyper-parameters that defined the network topology, i.e., the number of layers, type of layers (convolution, pooling, etc.), and order of the layers. We then applied parameters defined in the genes of the individual to construct and train a deep learning network on the MNIST dataset. The results of the network’s performance in testing were then used as the “fitness” of the individual in the EA population, i.e., individual networks that had high accuracy were considered to be the most fit. Typically, generating the results for a single network on a small dataset like MNIST requires a modest amount of GPU/CPU time, and memory. However, creating, training, and evaluating tens of thousands networks requires a significant number of GPUs, like those in the Titan high performance computer.

After all the individuals in the population were evaluated, the top performing individuals were selected to generate a new population of individuals that represented the next generation of the EA. These new generations contained a mix of the well performing hyperparameters from the best performing networks in the population. Successive generations of individuals gradually led to an improved set of hyperparameters over time. This method is called Multi-node Evolutionary Neural Networks for Deep Learning (MENNDL) [29].

For this experiment, we were looking to automatically discover hyperparameters of a well performing deep learning network on the MNIST dataset. We used a simple EA that limited the search to the number of neurons per layer and the kernel size of convolutional layers.

The network architecture utilized was LeNet [4] and featured two convolutional layers, two pooling layers, and one hidden fully-connected layer. This is the network that is most often used with the MNIST dataset in the literature.

We showed that even with this widely studied MNIST dataset, better hyper-parameters could be found than those widely reported in the literature. An EA that can evolve the topology provides the opportunity for improved results and the ability to process more challenging datasets. Such an EA also provides the opportunity to meaningfully utilize the entirety of Titan’s capacity. It provides challenging data management problems on a machine designed primarily for modeling and simulation, as opposed to these deep learning algorithms which require heavy amounts of data input in addition to heavy computation.

### Appendix B.2. Description of Neuromorphic Computing

A spiking neuromorphic approach to the MNIST problem was not the ideal solution since there is not a temporal component in the task of recognizing a handwritten digit. In order to leverage the temporal processing capabilities of spiking neural networks, we added a temporal component to the task by using a streaming scan of the digits as input to the SNN such that columns in the input image were received over time rather than all at once. The SNN learned to recognize digits based on this scan pattern. For the results presented on the MNIST task, the goal was to understand the deployment benefits of using an SNN in memristive hardware as opposed to classification accuracy on this problem. For the neutrino data, where the data itself already had a temporal component, there was a more natural mapping to SNNs. Thus, classification accuracy may be a more accurate representation of potential performance of SNNs in general than for non-temporal data like MNIST.

As noted in Appendix A.2, there are not very many SNN training methods or neuromorphic training methods that can be applied to spiking neuromorphic networks and operate within the characteristics and constraints of a particular neuromorphic hardware implementation. To train both SNN models and neuromorphic networks we utilized an evolutionary optimization (EO) approach to determine the structure (e.g., number of neurons and synapses and how they are connected) and parameters (e.g., weight values of synapses and threshold values of neurons) [51].

The neuromorphic system we used to explore both the MNIST and neutrino detection problem was a memristive implementation of the neuroscience-inspired dynamic architectures (NIDA) system [52]. NIDA is a simple SNN model composed of integrate-and-fire neurons and synapses with delays and weights that are affected by processes similar to long-term potentiation and long-term depression in biological brains. The NIDA model allows us to study neuromorphic models in software and determine how restrictions different in hardware (such as weight resolution or connectivity) affect performance.

The EO approach for training networks in the MNIST problem was previously applied to the NIDA SNN [52]. An ensemble approach was utilized where each network in the ensemble was responsible for recognizing a particular digit type. For example, a network may be trained to recognize zeros, in which case the network will take the handwritten digit image as input and its output corresponds to either “yes, it is a zero” or “no, it is not a zero”. Using this approach, ensembles that achieve around 90 percent accuracy were achieved.

The memristive device technology assumed for this simulation was characterized by a low resistance state (LRS) of 60 kΩ, about an order of magnitude larger than the resistance of a typical deep-submicron complementary metal–oxide–semiconductor (CMOS) transistor. This relatively high LRS for the memristor is desirable such that the CMOS channel resistance can effectively be neglected. The on-off ratio was assumed to be 10, providing a high resistance state (HRS) of 600 kΩ. Such characteristics for LRS, HRS and the associated on-off ratio have been observed for a range of memristive devices, including hafnium-oxide (HfO_2_) [53], tantalum-oxide (TaO_2_) [54], and titanium-oxide (TiO_2_) [55]. All of these memristive material stacks consist of an oxide layer sandwiched between two metallic layers. Depending on the polarity and magnitude of an applied voltage bias, the oxide layer transitions between being less or more conductive, providing the switching characteristics desirable for representing synaptic weights.

Our memristive NIDA simulation setup also included analog integrate-and-fire neurons, implemented using a 65 nm CMOS process technology. Neuromorphic elements (neurons and synapses) were simulated using Cadence Spectre and system-level energy and power estimates were calculated using a high-level simulator written in C++. Specifically, we verified the high-level C++ model versus the circuit level implementation using small networks that were simulated using both Cadence Spectre and the high-level NIDA simulator. Larger networks, specifically MNIST, were simulated using the high-level NIDA simulator to determine neuron and synapse activity information.

The memristive NIDA simulation was based on two significant steps. Initially an evolutionary optimization training process was used to generate optimized networks for the low level simulation. At the same time, the transistor level simulation was done using Cadence Spectre simulator. Estimates were collected for the design components in different conditions (neuron accumulating but not firing, neuron firing, etc.). These “per component” energy estimates were used in conjunction with activity information from the high-level NIDA simulation to calculate the total energy consumed.
